# Fullerenes against COVID-19: Repurposing C_60_ and C_70_ to Clog the Active Site of SARS-CoV-2 Protease

**DOI:** 10.3390/molecules27061916

**Published:** 2022-03-16

**Authors:** Tainah Dorina Marforio, Edoardo Jun Mattioli, Francesco Zerbetto, Matteo Calvaresi

**Affiliations:** Dipartimento di Chimica “Giacomo Ciamician”, Alma Mater Studiorum-Università di Bologna, Via Francesco Selmi 2, 40126 Bologna, Italy; edoardojun.mattioli2@unibo.it (E.J.M.); francesco.zerbetto@unibo.it (F.Z.)

**Keywords:** C_60_, C_70_, masitinib, M^pro^, SARS-CoV-2, COVID-19, drug repurposing, MM-GBSA, inhibitors, nanobio interface

## Abstract

The persistency of COVID-19 in the world and the continuous rise of its variants demand new treatments to complement vaccines. Computational chemistry can assist in the identification of moieties able to lead to new drugs to fight the disease. Fullerenes and carbon nanomaterials can interact with proteins and are considered promising antiviral agents. Here, we propose the possibility to repurpose fullerenes to clog the active site of the SARS-CoV-2 protease, M^pro^. Through the use of docking, molecular dynamics, and energy decomposition techniques, it is shown that C_60_ has a substantial binding energy to the main protease of the SARS-CoV-2 virus, M^pro^, higher than masitinib, a known inhibitor of the protein. Furthermore, we suggest the use of C_70_ as an innovative scaffold for the inhibition of SARS-CoV-2 M^pro^. At odds with masitinib, both C_60_ and C_70_ interact more strongly with SARS-CoV-2 M^pro^ when different protonation states of the catalytic dyad are considered. The binding of fullerenes to M^pro^ is due to shape complementarity, i.e., vdW interactions, and is aspecific. As such, it is not sensitive to mutations that can eliminate or invert the charges of the amino acids composing the binding pocket. Fullerenic cages should therefore be more effective against the SARS-CoV-2 virus than the available inhibitors such as masinitib, where the electrostatic term plays a crucial role in the binding.

## 1. Introduction

The onslaught of COVID-19 waves and the continuous rise of variants of the SARS-CoV-2 virus [1] demand new treatments to complement vaccinations. Endocytic entry into host cells, RNA replication and transcription, translation and proteolytic processing of viral proteins, virion assembly, and release of new viruses through exocytic mechanisms are all potentially targetable processes in the coronavirus life cycle [2].

Among the viral proteins, only a few are essential in the life cycle of the virus. The main protease, known as M^pro^ or 3CL^pro^, plays a critical function in viral replication and transcription and represents the main target for medicinal chemistry [3,4]. This enzyme breaks down the polyprotein chain coded by the RNA of the virus into functional proteins, which the virus needs to construct itself and proliferate [3,4]. Disrupting this important part of the virus’s self-replication engine blocks the infection.

**Figure 1:**
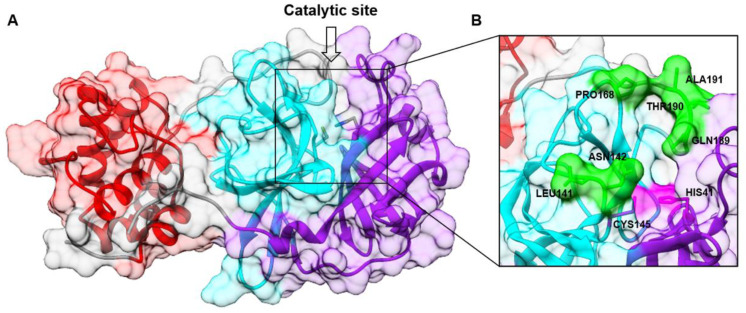
(**A**) M^pro^ structure (PDB ID 7JU7) shown in ribbons and surface representation: domain I (residues 8–101) in purple, domain II (residues 102–184) in cyan, and domain III (residues 201–303) in red. (**B**) A magnification of the catalytic site showing the catalytic dyad (His41 and Cys145) in purple and residues that lie within the binding pocket in green.

Just a few weeks after the first COVID-19 outbreak, the crystallographic structure of M^pro^ was determined and deposited under the PDB code 6LU7 [3]. M^pro^ is composed of 306 amino acids, characterized by 3 distinct domains (domains I, II, and III) [3]. Domain I (residues 8–101) and domain II (residues 102–184) have a similar fold composed of antiparallel ß-barrel structures. Domain III (residues 201–303), instead, consists of a cluster of five α-helices, responsible for protein dimerization. The active site is placed in a cleft between domains I and II (Figure 1). The catalytic residues Cys145 and His41 are buried in this cavity that can accommodate four substrate residues in positions P1′ through P4 and is flanked by residues from both domains I and II (Figure 1B). The catalytic dyad may be activated by a proton transfer from Cys145 to His41, possibly triggered by substrate binding or occurring in a transition state during the attack by the sulfur on the carbonyl carbon atom of the scissile peptide bond. It was suggested that a water molecule might complete the catalytic triad by mediating crucial interactions between His41 and other important conserved residues, such as His164 and Asp187 [4].

Due to the immediate availability of the M^pro^ crystal structure, structure-based drug discovery (SBDD) techniques were promptly used to expedite the rational identification of potential M^pro^ inhibitors [5,6,7] or to drive the repurposing of known molecules [8,9,10,11,12,13,14]. Many protease inhibitors of the human immunodeficiency virus (HIV) were identified as possible anti-COVID candidates [15].

Fullerenes and carbon nanomaterials are able to interact with peptides [16,17] and proteins [18,19,20,21,22,23,24,25,26] and, in general, are considered promising antiviral agents [27,28,29,30,31,32]. The idea of using C_60_ as an inhibitor of the HIV protease dates back to 1993 [33]. C_60_ inhibits the protein thanks to its size and unusual spherical shape [33]. The buckyball fits snugly into the substrate binding pocket, blocks the active site, and prevents the HIV polypeptide chain from entering [33]. C_60_ is, however, insoluble in water, and fullerene derivatives were designed and then synthesized for use in a physiological environment [34,35,36,37,38,39,40,41,42,43,44]. The structure–activity relationship between functionalized fullerenes and HIV protease inhibition showed the importance of positioning the derivative moieties in a well-defined geometry on the fullerene cage [34,35,36,37,38,39,40,41,42,43,44]. 

The idea of repositioning C_60_ for the inhibition of SARS-CoV-2 M^pro^ is a natural consequence [45,46,47]. In this work, we compare (i) the binding energy of C_60_ with the HIV protease and with SARS-CoV-2 M^pro^ to understand the efficiency of the repurposing, and (ii) the performances of C_60_ and masitinib [48], a known inhibitor of SARS-CoV-2 M^pro^, to verify the possibility of using C_60_ derivatives as effective M^pro^ inhibitors. We further propose, for the first time, the use of C_70_ as an innovative scaffold for the inhibition of SARS-CoV-2 M^pro^.

## 2. Results

### 2.1. Determining the C_60_ Binding Pocket in SARS-CoV-2 M^pro^

**Figure 2:**
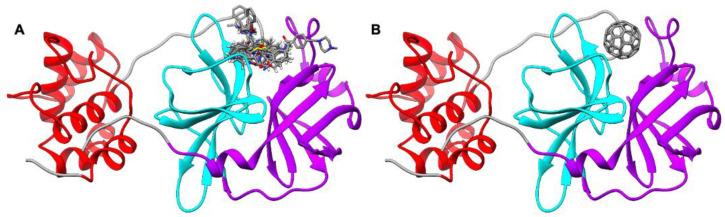
(**A**) Binding of M^pro^ ligands N3 (PDB ID 6LU7), masitinib (PDB ID 7JU7), parampanel analogue compound 5 (PDB ID 7L11), MPI4 (PDB ID 7JQ1), ML188 (PDB ID 7L0D), isofloxythepin (PDB ID 7AY7), carmofur (PDB ID 7BUY), UAW243 (PDB ID 6XFN), and boceprevir (PDB ID 6XQU) in M^pro^. The protein structure is shown with the ribbon. (**B**) Identification of the fullerene binding pocket in M^pro^.

The crystal structures of SARS-CoV-2 M^pro^ complexed with different ligands (Figure 2A) showed that noncovalent and covalent inhibitors behave differently. Noncovalent inhibitors interact with the multiple residue system of the substrate binding pocket of SARS-CoV-2 M^pro^. Covalent inhibitors mainly interact with the catalytic Cys145 residue after an initial noncovalent interaction in the substrate binding pocket. 

Using a docking protocol able to identify the fullerene binding pockets of proteins [18,19,49,50,51,52,53,54], we docked the C_60_ in SARS-CoV-2 M^pro^. C_60_ binds on the substrate binding pocket of M^pro^ (Figure 2B). The fullerene cage shows a strong shape complementarity with this pocket of M^pro^. C_60_ occupies exactly the same position occupied by known M^pro^ inhibitors, suggesting an inhibitory activity of the cage.

### 2.2. Determining the Binding Energy between C_60_ and SARS-CoV-2 M^pro^

The atomistic understanding of the interactions of C_60_ with SARS-CoV-2 M^pro^ is crucial for real applications of nanomolecules in medicine [26]. MD simulations represent a powerful tool to investigate such interactions [55]. Starting from the docking pose, 100 ns of molecular dynamics simulations was carried out. To estimate the binding energy between M^pro^ and C_60_, an MM-GBSA analysis of the trajectories was performed. C_60_ lies above His41, giving sandwich-like π–π interactions [56,57], and interacts hydrophobically [56,57] with Met49 and Leu27. Surfactant-like interactions [56,57] with Cys145 and Ser46 are also observed. 

The ΔG_binding_ between M^pro^ and C_60_ is −18.8 kcal mol^–1^. This value is very close to the interaction energy between lysozyme and C_60_ (−18.5 kcal mol^−1^) [50], a complex that is experimentally accessible and widely used in nanomedicine [49,58,59,60]. The result demonstrates the feasibility of the exploitation of the C_60_ molecule in the inhibition of M^pro^.

According to the analysis of the binding components of the energy, the driving force for binding (−44.8 kcal mol^–1^) is represented by van der Waals interactions. Hydrophobic interactions, (E_non-polar_), assist the binding, despite the fact that their value (−2.5 kcal mol^–1^) is far lower than that of the vdW interactions. The contributions of polar solvation (12.8 kcal mol^–1^) and entropy (15.7 kcal mol^−1^) are positive and oppose the binding. Because of the rigidity of CNPs, the entropic term is frequently overlooked [45] while studying protein–CNP interactions. However, this factor, which is estimated to be 15.7 kcal mol^–1^, is energetically significant and should be considered when protein–CNP hybrids are studied. The binding of C_60_ to the protein cavity produces a significant reduction in amino acid mobility, giving rise to this high value.

**Figure 3:**
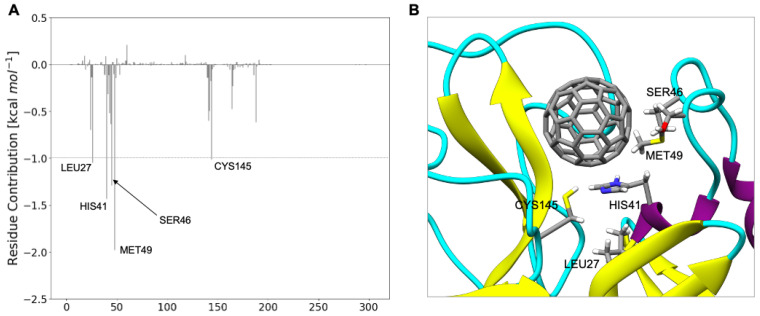
(**A**) C_60_@M^pro^ interactions. ΔG_binding_ decomposed *per* residue. (**B**) Interaction between Met49, His41, Ser46, Leu27, and Cys145 and C_60_.

The decomposition analysis of the total binding energy provides the contribution to the binding of each amino acid (Figure 3). The most interacting amino acids (∆G_binding_ larger than 1.0 kcal mol^−1^) are Met49, His41, Ser46, Leu27, and Cys145. Very interestingly, C_60_ strongly interacts both with the catalytic dyad (Cys145-His 41) and with residues located on the substrate binding pocket (Met49, Ser46, Leu27).

At the same time, C_60_ shields the catalytic dyad, blocking its catalytic activity, and occupies the substrate binding pocket, impeding the interaction of M^pro^ with its substrate. 

### 2.3. Comparing the Binding of C_60_ in SARS-CoV-2 M^pro^ and HIV Protease

To validate the results and evaluate the reliability of the use of C_60_ as an M^pro^ inhibitor, we calculated the interaction of C_60_ with the HIV protease (Pro_HIV_), using the same protocol adopted to calculate the binding energy between C_60_ and M^pro^ (Table 1).

**Figure 4:**
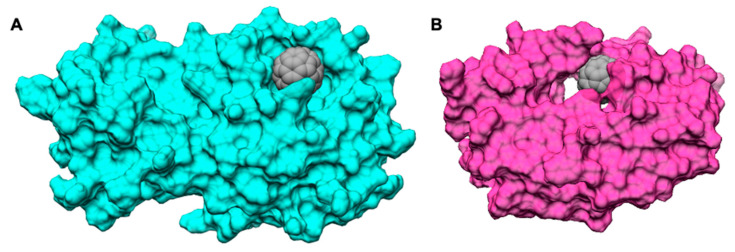
Surface complementarity between (**A**) M^pro^ and (**B**) Pro_HIV_ proteins and the C_60_ cage.

Shape complementarity is the crucial parameter governing the interaction of fullerene with proteins [57]. In Pro_HIV_, as well as in M^pro^, C_60_ fits snugly in the active site (Figure 4), and as a consequence, the total binding energy and the energy components of ΔG_binding_ between C_60_ and the two proteases are similar [61]. 

Since it is known that C_60_ experimentally works as an HIV protease inhibitor [33,34,35,36,37,38,39,40,41,42,43,44], this comparison validates the idea of repurposing C_60_ as a SARS-CoV-2 M^pro^ inhibitor.

### 2.4. Comparing the Binding of C_60_ in SARS-CoV-2 M^pro^ with Masitinib

**Figure 5:**
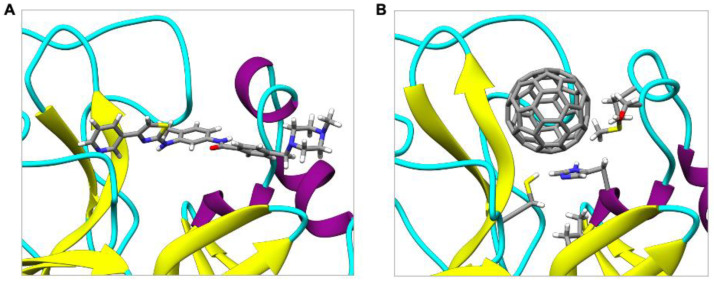
Binding of (**A**) masitinib and (**B**) C_60_ with M^pro^.

To estimate the potential application of C_60_ as an inhibitor of M^pro^, we calculated the binding energy with a known inhibitor of M^pro^, namely, masitinib. Masitinib is an orally bioavailable tyrosine kinase inhibitor, repurposed as an inhibitor of SARS-CoV-2 M^pro^ [48]. X-ray crystallography and biochemistry experiments showed that masitinib acts as a competitive inhibitor of M^pro^ [48] and occupies the same binding pocket of C_60_ (Figure 5). Mice infected with SARS-CoV-2 and subsequently treated with masitinib had a 200-fold decrease in viral titers in the lungs and nose, as well as lower lung inflammation [48]. 

The results show that C_60_ has a larger binding energy than masitinib, due to two factors that are usually ignored when the interactions between inhibitors and proteins are evaluated, namely, desolvation energy and entropy (Table 2). 

Even if vdW and electrostatic terms are larger for masitinib than for C_60_, binding of the more polar and more flexible masitinib molecule gives larger penalty terms due to the desolvation energy and entropy. C_60_ is an ideal inhibitor because the terms that generally oppose binding (i.e., desolvation energy and entropy) are minimized by its hydrophobicity and rigidity.

### 2.5. Determining the Binding Energy between C_70_ and SARS-CoV-2 M^pro^

**Figure 6:**
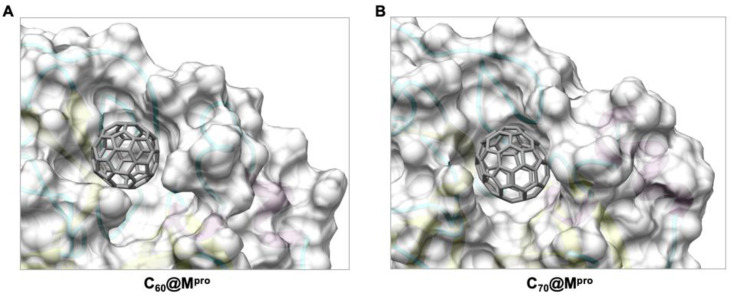
Surface representation of the binding cleft of (**A**) C_60_ and (**B**) C_70_ in M^pro^.

With the increase in the carbon cage size, the binding strength between proteins and fullerenes usually increases [60,62]. It is natural to suppose that C_70_, in principle, may be a better inhibitor than C_60_ for M^pro^. Docking calculations showed that C_70_ occupies the same binding pocket (Figure 6).

The ΔG_binding_ between C_70_ and M^pro^ is considerably higher (−28.0 kcal mol^−^^1^) than in the case of C_60_ (−18.8 kcal mol^−^^1^) (Table 3). 

**Figure 7:**
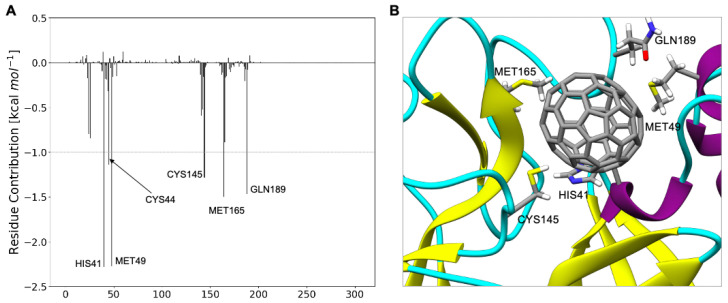
(**A**) C_70_@M^pro^ interactions. ΔG_binding_ decomposed *per* residue. (**B**) Interaction between His41, Cys44, Met49, Cys145, Met165, and Gln189 and C_70_.

This increase is due to a substantial increase in the van der Waals interactions, which grow from −44.8 kcal mol^−^^1^ for C_60_ to −59.2 kcal mol^−^^1^ for C_70_. Van der Waals and hydrophobic interactions are −47.3 kcal mol^−^^1^ in C_60_ and −62.4 kcal mol^−^^1^ in C_70_, with a net increase of 15.1 kcal mol^−^^1^. In C_70_, the increase compensates the small energy penalties due to higher entropic and desolvation terms (28.5 kcal mol^−^^1^ in C_60_ and 34.4 kcal mol^−^^1^ in C_70_, with a net increase of 5.9 kcal mol^−^^1^). The measure of shape complementarity between the fullerene cage and the protein is usually taken as a quick way to estimate the stabilizing interactions. This is also true in this case considering that the variation in the solvent-accessible surface area (∆SASA) upon binding is 347.2 Å^2^ for C_60_@M^pro^ and 444.4 Å^2^ for C_70_@M^pro^, as evident also in Figure 6. The larger C_70_ engages stronger interactions with a larger number of the amino acids that make up the binding pocket (Figure 7). The interactions of C_60_ with the catalytic dyad of His41 and Cys145 are −1.4 kcal mol^−^^1^ and −1.0 kcal mol^−^^1,^ respectively. The interactions of C_70_ with the same residues are −2.3 kcal mol^−^^1^ and −1.3 kcal mol^−^^1^, with an increase of 0.9 kcal mol^−^^1^ and 0.3 kcal mol^−^^1^. In addition, C_70_ also strongly interacts with Met165 (−1.5 kcal mol^−^^1^) and Gln189 (−1.5 kcal mol^−^^1^), residues that were identified as the hot spot for the activity of M^pro^ inhibitors [63].

### 2.6. Evaluating the Binding Enrgy of Masitinib, C_60_, and C_70_ to SARS-CoV-2 M^pro^ in Different Protonation States of the Catalytic Dyad

The protonation state of histidine [64,65,66,67], and in particular of the catalytic dyad (His41-Cys145), plays a crucial role in M^pro^ activity, stability, and protein–ligand interactions. Very recently, neutron crystallographic studies [65,67] provided direct visualization of the hydrogen atoms’ locations in the SARS-CoV-2 M^pro^ enzyme. If in the bound state His41 is in a neutral form [65], in the ligand-free structure, the catalytic site of M^pro^ adopts a zwitterionic form where Cys145 is deprotonated and negatively charged and His41 is doubly protonated and positively charged [67]. Since the free energy of activation for the initial proton transfer from Cys145 to His41 is very low [68,69] and the relative energies of the zwitterionic and neutral states are very close [68,69], we reinvestigated the binding of masitinib, C_60_, and C_70_ to M^pro^ in both protonation states of the catalytic dyad (Table 4).

The protonation state of the catalytic dyad strongly affects the binding of masitinib, and in the zwitterionic form (His41^+^-Cys145^−^), the ∆G_binding_ is markedly reduced (going from −15.5 kcal mol^−^^1^ to −5.1 kcal mol^−^^1^). The electrostatic term decreases from −18.1 kcal mol^−^^1^ for the doubly neutral dyad to −11.0 kcal mol^−^^1^ for the zwitterionic dyad, due to the different distributions of the charges in the binding site. This term is unaffected in the fullerene binding because it does not have net charges. Owing to increased vdW interactions, ∆G_binding_ of fullerenes increases in the case of the zwitterionic form of the dyad. It goes from −44.8 kcal mol^−^^1^ to −54.6 kcal mol^−^^1^ for C_60_ and from −59.2 kcal mol^−^^1^ to −60.9 kcal mol^−^^1^ for C_70_. 

## 3. Conclusions

In summary, we demonstrated the possibility of repurposing fullerenes as inhibitors of SARS-CoV-2 M^pro^. We calculated and compared the binding energies of (a) C_60_ and C_70_ to M^pro^, (b) C_60_ to the HIV protease, and (c) masitinib to M^pro^. The results indicate the feasibility of the repurposing. Fingerprint analysis showed the role of π–π, hydrophobic, and surfactant-like interactions in the binding of the fullerenes to M^pro^. Fullerenes interact with the His41-Cys145 dyad, blocking the catalytic activity. At the same time, they occupy the substrate binding pocket, impeding the interaction of M^pro^ with its substrate. 

Fullerenes are ideal inhibitors because, in these molecules, the terms that generally oppose the binding (i.e., desolvation energy and entropy) are minimized by their hydrophobicity and rigidity.

Shape complementarity is crucial to govern the interaction of fullerenes with proteins, and also in this case, the larger C_70_ interacts more strongly than C_60_ with the binding site of M^pro^. C_70_ engages stronger interactions with more residues that form the binding pocket. In particular, C_70_ strongly interacts with the Met165 and Gln189 residues that were identified as the hot spot for the activity of M^pro^ inhibitors.

Fullerenes are insensitive to mutations that perturb the electrostatic characteristics of the binding site. They interact even more strongly with M^pro^ when different protonation states of the catalytic dyad are considered. 

The binding of fullerenes to M^pro^, based as it is on shape complementarity, i.e., vdW interactions, is aspecific. As such, it is not sensitive to mutations that eliminate or invert the charges of the amino acids composing the binding pocket. Indeed, for some inhibitors, such as masinitib, the electrostatic terms play a crucial role. As a consequence, even a punctual modification of the catalyic site may strongly affect their binding, and therefore their activity. Fullerenes therefore appear to be ideal moieties to exploit in the identification of potential new drugs against the SARS-CoV-2 protease and other viral proteins.

## 4. Materials and Methods

### 4.1. System Setup 

The crystal structures of the SARS-CoV-2 main proteases (PDB ID 6LU7, 7JU7, and 7L10) and the HIV protease (PDB ID 1ZTZ) were downloaded from the Protein Data Bank (PDB). The crystal structure of PDB ID 7JU7 was used for the docking calculations.

The Amber ff14SB force field [70] was used to model the M^pro^ and Pro_HIV_ proteins. C_60_ and C_70_ carbon atoms were modeled as uncharged Lennard-Jones particles by using sp2 carbon parameters taken from the ff14SB force field [70]. Masitinib was parametrized by calculating the partial atomic charges using the restraint electrostatic potential method (RESP) at the HF/6-31G* level of theory. The corresponding parameters were then generated by the standard procedure reported for antechamber, as implemented in Amber16 [71]. 

### 4.2. Docking

Docking models were obtained using the PatchDock algorithm [72] that computes the shape complementarity between two entities (ligand and receptor), minimizing the number of steric clashes. PatchDock (i) assigns concave, convex, or flat patches to the ligand and receptor surface, (ii) matches concave–convex/flat–flat, and generates a set of candidate transformations. (iii) Each transformation is then ranked by the shape complementarity and the atomic desolvation energy of the complex (scoring functions). Root mean square deviation clustering avoids the generation of redundant solutions. 

### 4.3. MD Simulations

The docking structures were minimized by 5000 steps of steepest descent minimization, followed by 5000 steps of the conjugate gradient algorithm. The minimized structures underwent a 1 ns equilibration step and were heated from 0 to 300 K (Langevin thermostat). Periodic boundary conditions (PBC) and particle mesh Ewald summation were used throughout (with a cut-off radius of 10 Å for the direct space sum). The MD simulations were carried out using an explicit solvent (TIP3P water model). Sodium counterions were included to exactly neutralize the charge of the system. After the equilibration, a production MD simulation of 100 ns was carried out for every system at 300 K. Amber 16 was used to run all the simulations [71]. 

### 4.4. Molecular Mechanics/Generalized Born Surface Area (MM/GBSA) Analysis

A total of 5000 frames were extracted from MD simulations and used for the MM-GBSA analysis. An infinite cut-off was used for all the interactions. The electrostatic contribution to the solvation free energy was calculated with the Generalized Born (GB) model, as implemented in MMPBSA.py [73]. The nonpolar contribution to the solvation free energy was determined with solvent-accessible surface-area-dependent terms. To obtain an estimate of the binding entropy, the normal modes for the complex, receptor, and ligand were calculated, and the results were averaged using the PTRAJ program (Normal Mode Analysis) via MMPBSA.py [73].

## Figures and Tables

**Table 1:** Energy components of ΔG_binding_ (VDW, E_el_, E_GB_, and E_non-polar_) for C_60_@M^pro^ and C_60_@Pro_HIV_ complexes. All energies are reported in kcal mol^−1^.

Complex	ΔH	VDW	E_El_	E_GB_	E_non-polar_	TΔS	ΔG_bind_
C_60_@M^pro^	−34.5	−44.8	0.0	12.8	−2.5	−15.7	−18.8
C_60@_Pro_HIV_	−38.0	−51.6	0.0	16.0	−2.4	−18.3	−19.7

**Table 2:** Energy components of ΔG_binding_ (VDW, E_el_, E_GB_, and E_non-polar_) for C_60_@M^pro^ and masitinib@M^pro^ complexes. All energies are reported in kcal mol^−1^.

Complex	ΔH	VDW	E_El_	E_GB_	E_non-polar_	TΔS	ΔG_bind_
C_60_@M^pro^	−34.5	−44.8	0.0	12.8	−2.5	−15.7	−18.8
masitinib@M^pro^	−41.2	−51.4	−18.1	34.0	−5.8	−24.7	−16.5

**Table 3:** Energy components of ΔG_binding_ (VDW, E_el_, E_GB_, and E_non-polar_) for C_60_@M^pro^ and C_70_@M^pro^ complexes. All energies are reported in kcal mol^−1^.

Complex	ΔH	VDW	E_El_	E_GB_	E_non-polar_	TΔS	ΔG_bind_
C_60_@M^pro^	−34.5	−44.8	0.0	12.8	−2.5	−15.7	−18.8
C_70_@M^pro^	−45.6	−59.2	0.0	16.8	−3.2	−17.6	−28.0

**Table 4:** Comparison of the binding energy of masitinib, C_60_, and C_70_ with M^pro^ in the two protomeric states, namely, His41-Cys145 and His41^+^-Cys145^−^. All energies are reported in kcal mol^−1^.

Complex	His41-Cys145 ΔG_binding_	His41^+^-Cys145-ΔG_binding_
masitinib@M^pro^	−16.5	−5.1
C_60_@M^pro^	−18.8	−24.3
C_70_@M^pro^	−28.0	−29.4

## Data Availability

All data in this study can be requested from the corresponding authors (tainah.marforio2@unibo.it, T.D.M.; matteo.calvaresi3@unibo.it, M.C.).
